# Distance Entropy Cartography Characterises Centrality in Complex Networks

**DOI:** 10.3390/e20040268

**Published:** 2018-04-11

**Authors:** Massimo Stella, Manlio De Domenico

**Affiliations:** Fondazione Bruno Kessler, Via Sommarive 18, 38123 Povo, Italy

**Keywords:** complex networks, network measures, entropy, closeness centrality, multiplex lexical networks

## Abstract

We introduce distance entropy as a measure of homogeneity in the distribution of path lengths between a given node and its neighbours in a complex network. Distance entropy defines a new centrality measure whose properties are investigated for a variety of synthetic network models. By coupling distance entropy information with closeness centrality, we introduce a network cartography which allows one to reduce the degeneracy of ranking based on closeness alone. We apply this methodology to the empirical multiplex lexical network encoding the linguistic relationships known to English speaking toddlers. We show that the distance entropy cartography better predicts how children learn words compared to closeness centrality. Our results highlight the importance of distance entropy for gaining insights from distance patterns in complex networks.

## 1. Introduction

Network science provides a powerful framework for modelling and understanding how individual entities give rise to complex—and often unexpected—phenomena by interacting with each other [1,2,3,4,5,6]. Network models encapsulate the topology of interactions among entities by means of links distributed among nodes. Defining the centrality of nodes in complex networks is an important question for determining the role of individual agents in a variety of dynamical processes such as information flow and influence maximisation [7,8,9], network growth [1], and resilience to cascade failures [10,11].

In many of these processes, centrality can be defined by means of network distance—that is, the minimum number of links separating any two nodes. Overwhelming evidence from real-world network analysis has shown how distance among nodes is an important indicator of the evolution of a given process: in general, information flows at slower rates between nodes at greater distance in social networks [7]; the recollection of words at greater distance in semantic networks in memory tasks is slower [12]; smaller network distance between oscillators facilitates synchronization [9] and consensus [13]; and there is a higher turnover rate of animal and plant species among closer sites in river networks [14].

Network distance can be used to quantify a node’s centrality in spreading processes where the flow follows shortest paths and such centrality is called closeness [7,15]. Closeness centrality quantifies the average distance of all the network paths leading to a given node. In undirected, unweighted networks, closeness centrality $c_{i}$ of node *i* (i=1,2,…,N) is defined as [15]:

(1) $$c_{i}=\frac{C_{i}}{\sum_{j\neq i}d_{ij}},$$

where $C_{i}$ is the number of nodes in the same connected component of *i* and $d_{ij}$ is the network distance between nodes *i* and *j*. It is worth noting that $C_{i}=N$ for networks with a single connected component. It has been shown that this estimator is ill-defined in the case of disconnected networks [16]; however, in the following we will deal only with connected networks and we can safely use the above definition of closeness centrality.

Closeness centrality has proved to be powerful in many real-world applications [7,11,17,18,19]. Recently, the closeness centrality of words in multiplex lexical networks of word–word similarities resulted as an optimal predictor of word learning [18], outperforming other single- and multi-layer network measures such as degree, betweenness, local clustering, PageRank, eigenvector centralities, and even word statistical features such as frequency and word length. In this work, we enrich closeness centrality with a new measure of *distance entropy*, quantifying node centrality through the distribution of path lengths. We develop our analysis within the established framework of graph distance-based entropies [20,21,22,23], which represent information-theoretic measures for characterising structural patterns in graphs. We test the combination of distance entropy and closeness centrality—a composition we call *distance entropy cartography*—in artificial network models and then apply it to improving word prediction based on closeness only. Distance entropy significantly improves the already optimal results of closeness for the prediction of word learning. These improvements are not observed when closeness is coupled with other centrality measures such as degree. Our results provide compelling evidence for the importance of considering information measures relative to shortest paths for gaining insights into real-world complex systems.

## 2. Introducing a New Distance Entropy

The definition of a graph entropy for a network *G* with *N* nodes relies on the choice for an information functional $f_{i}$ to attribute to each node (e.g., degree centrality). The information functional determines a node probability $p_{i}=f_{i}/\sum f_{i}$ for each node *i*, and the relative graph entropy is defined as the Shannon entropy of the probabilities $p_{i}$ (i=1,2,…,N) [20].

Closeness centrality represents the inverse of the mean value of the distribution of path lengths from a given node to the rest of the system. Hence, closeness alone offers no information about the spread of the distribution of network distances. To account for this spread, we introduce the *distance entropy* h(i), defined as the information entropy of the set $d^{\left(i\right)}\equiv \left(d_{i1},\ldots ,d_{ij},\ldots ,d_{iN}\right)$ of distances between node *i* and any other node *j* in the system, here assumed to be of size *N* (1≤j≤N). Let us denote with $M_{i}$ the maximum distance $M_{i}=\max_{j}d_{ij}$ and with $m_{i}$ the minimum distance $m_{i}=\min_{j}d_{ij}$. Let us denote by $p_{k}^{\left(ij\right)}=P\left(d_{ij}=k\right)$ with $m_{i}\leq k\leq M_{i}$, the probability that the generic entry $d_{ij}$ is equal to *k*. If node *i* is at distance *k* from $n_{k}$ other nodes, then $p_{k}^{\left(ij\right)}=n_{k}/\left(N-1\right)$. In the graph entropy literature, $n_{k}$ represents our chosen information functional. Chen et al. [21] considered the same information functional, but they limited themselves to considering only nodes of a given specific distance *k* from node *i*, at variance with our approach considering all possible distances ($m_{i}\leq k\leq M_{i}$). Other approaches used distances as information functionals, but did not consider counting paths of a given length (for a review, we refer the interested reader to [20,23]). We exclude the distance of a node from itself. In this analysis, we introduce the following definition of distance entropy as:

(2) $$h\left(i\right)=-\frac{1}{\log(M_{i}-m_{i})}\sum_{k=1}^{M_{i}-m_{i}}p_{k}^{\left(i\right)}\log p_{k}^{\left(i\right)}.$$

With this definition, h(i) ranges between 0 and 1. Note that our definition can also be generalised to strongly connected components [2] of directed networks by counting lengths in directed paths. The extension to weighted networks is not trivial, as the definition of shortest paths in this case is not unique [24,25,26]. Here we explore the case of undirected, unweighted graph models and real-world networks.

It is worth remarking that we can interpret the meaning of the extremal values of our distance entropy in terms of network centrality by considering specific classes of regular graphs, as in the following.

### Distance Entropy in Regular Graphs

Let us consider a complete graph of *N* nodes, $K_{N}$. In this graph, node *i* is at distance 1 from all the other nodes, so then $d^{\left(i\right)}=\left(1,\ldots ,1\right)$ is a set of N−1 entries, all equal to 1. It is straightforward to verify that, in this case, the information entropy for node *i* is h(i)=0. The same analysis holds for all the other N−1 nodes in the complete graph. Hence, all the nodes in a complete graph have distance entropy h=0. More generally, distance entropy is 0 for all nodes adjacent to all other nodes in any given network.

In star graphs, there is a central node connected to all other peripheral nodes and no other links are present. The result for nodes in complete graphs also holds for the star centre. Consequently, the centres of star graphs have distance entropy h=0. Hence, we can interpret distance entropy as a measure of the regularity of the distribution of path lengths between a given node and its neighbours, with h=0 representing the case of maximum homogeneity in the path length distribution. Since it is not possible for a node to be at distance d≥2 from all other nodes simultaneously in a connected network, then h=0 identifies nodes adjacent to all other nodes.

In a ring graph where N>3 nodes have only two neighbours, then every node has the same set of distances to the other N−1 nodes and the possible distances are $1,2,\ldots ,\left\lfloor N/2\right\rfloor$, where $\left\lfloor .\right\rfloor$ is the floor function. If *N* is odd, then it is easy to check that $p_{k}=2/\left(N-1\right)$. Consequently, the entropy of any node *i* in a ring graph with an odd number of nodes is:

(3) $$h\left(i\right)=-\frac{\left\lfloor N/2\right\rfloor }{\log\left\lfloor N/2\right\rfloor }\left(\frac{2}{N-1}\right)\log\left(\frac{2}{N-1}\right)=-\frac{1}{\log[(N-1)/2]}\log\left(\frac{2}{N-1}\right)=1.$$

Hence, when *N* is odd, then all nodes in a ring graph have maximum distance entropy h=1, which corresponds to the case of minimum homogeneity of path lengths; that is, paths between connected nodes have lengths that are distributed uniformly across all possible distances.

When *N* is even, then $p_{k}=2/\left(N-1\right)$ except for k=N/2, for which $p_{N/2}=1/\left(N-1\right)$. Then the formula for the distance entropy of a node becomes:

(4) $$h\left(i\right)=-\frac{1}{\log(N/2)}\left[\frac{N-2}{N-1}\log\left(\frac{2}{N-1}\right)+\frac{1}{N-1}\log\left(\frac{1}{N-1}\right)\right].$$

Note that ring graphs are lattice graphs for which the coordination number z=2 (i.e., every node is connected only to two other nodes). While the analytical results for ring graphs can also be extended to cases for z>2, when *z* approaches N−1 the lattice becomes a complete graph and hence h→0 for every node. When z≪N−1, instead, results similar to the ring lattices hold and the lattice nodes are expected to have values of distance entropy close to 1. Rather than considering other regular structures, we now focus on characterising patterns of distance entropy in network models frequently used in the relevant literature.

## 3. Distance Entropy and Network Models

We consider three main network models usually considered in the literature: Erdos–Renyi (ER) random graphs [27], Watts–Strogatz small-world (SW) networks [28], and Barabasi–Albert (BA) preferential attachment networks [1]. For each network model, we are interested in characterising trends of the average distance entropy depending on the model parameters. In one case, we discover the presence of a tipping point for distance entropy in ER random graphs and relate it numerically to the appearance of short-cuts for increasing edge densities.

### 3.1. Homogeneous Random Graphs

**Figure 1:**
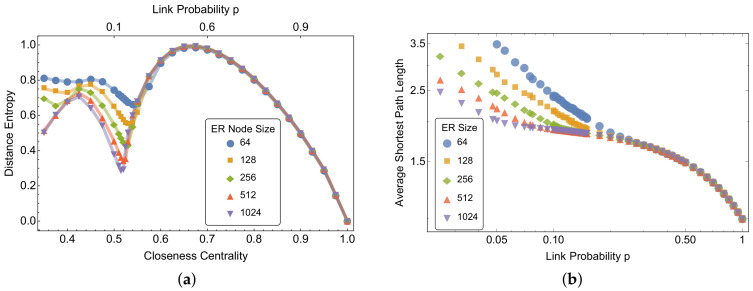
(**a**) Mean closeness centrality and distance entropy of Erdos–Renyi (ER) random graphs of different node sizes and different link probabilities. Mean values are averaged for all nodes in a graph and across 100 different graph realisations. Link probabilities are relative to all node sizes. All different ensembles converge to the same pattern of distance entropy roughly above rewiring probability p=0.6. (**b**) On a log–log scale, average shortest path length of ER random graphs for different link probabilities. The average distance decreases with increasing rewiring probability *p* and tipping points are evident around p≈0.1, after which the average shortest path length decreases with a slower rate.

Figure 1a plots the mean closeness centrality and distance entropy of ER random graphs of different size *N* and different link probability *p*. As expected, the addition of links makes random graphs closer to complete graphs, increasing the average closeness centrality when *p* rises. Instead, numerical results on the distance entropy indicate that the entropy of path lengths in ER random graphs is not monotonic: we identify a tipping point—depending on system size—in the average entropy that asymptotically converges to $p^{*}\approx 0.1$, which is well above the critical values for the emergence of the largest connected component $p_{c}=1/N$ and for connectedness $p_{c}=\log N/N$. A thorough characterization of this tipping point, identifying a structural change of paths in ER random graphs, is outside of the scope of the present work. It is interesting to note that all of the simulated ensembles converge towards the same pattern of distance entropy and closeness for increasing values of $p>p^{*}$, independently of their size. Before the tipping point $p<p^{*}$, short-cuts (i.e., paths with length 1) start appearing in the networks when *p* approaches $p^{*}$ from the left, as also noticeable from the trend of average shortest path length on *p* (see Figure 1b). Once created, short-cuts make nodes closer to each other, path lengths of shorter length start appearing with higher frequency, and hence the average distance entropy reduces. However, the densification due to increases in *p* happens at random, and hence not all possible short-cuts are created in the network when *p* is slightly higher than $p^{*}$. The random occurrence of edges will give rise to shortest paths having a homogeneous length distribution corresponding to increases in the values of *h* up to values close to 1. When even more links are added, the random graphs get closer to a complete graph, for which h=0, and hence the average distance entropy decreases.

### 3.2. Small-World Networks

**Figure 2:**
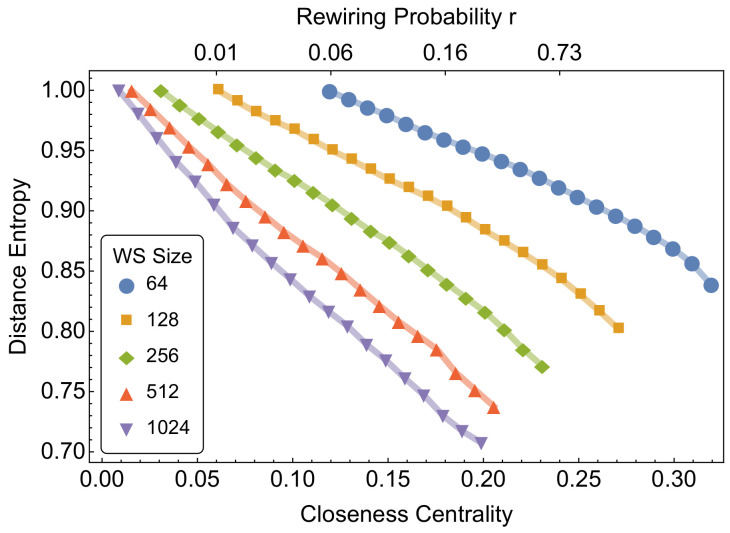
Mean closeness centrality and distance entropy of Watts–Strogatz small-world (SW) networks of different node sizes and different rewiring probabilities. The rewiring probabilities plotted above are relative only to the case with N=256, and are provided only as a guideline. Mean values are averaged for all nodes in a graph and across 100 different independent realizations.

Figure 2 reports the patterns of mean closeness centrality and distance entropy for small-world networks of different sizes, for coordination number z=4 and at different rewiring probabilities *r*. When r=0, a small-world network is a lattice with coordination number *z*. For intermediate values 0<r<1, a fraction *r* of the links for each node is rewired uniformly at random. For r=1, a small-world network is equivalent to a random graph with p=z/N [28].

Figure 2 shows that the rewiring probability has a monotonic effect on the mean distance entropy, which decreases from values close to 1 (r=0). This is expected, as the rewiring is increasingly destroying the order of the lattice structure, thus reducing *h*. Note that the minimum value of distance entropy—reached when r=1—is the same mean distance entropy of a random graph with p=z/N. The numerical results in Figure 2 indicate that distance entropy does not detect the so-called small-world regime (i.e., a region determined by intermediate values of *r* for which small-world display an average short path length close to logN and values of clustering coefficient comparatively higher than those of a random graph). In fact, no tipping points relative to this phase transition are found in the numerical simulations, independently of the considered network size. We attribute this finding to the fact that distance entropy can only highlight deviations from a homogeneous distribution of path lengths, and cannot provide information about either the assortative mixing or the clustering of nodes, which are both network features that must be measured in order to characterise the small-world property.

### 3.3. Barabasi–Albert Networks

**Figure 3:**
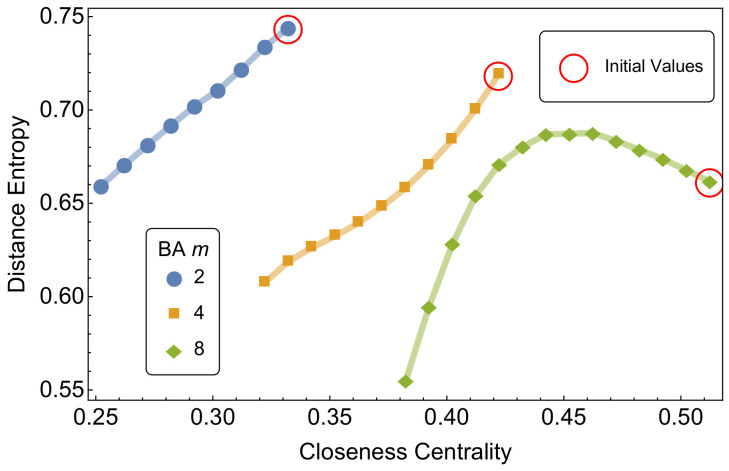
Mean closeness centrality and distance entropy of *growing* Barabasi–Albert (BA) networks for different values of the link growth rate *m*. Initial values are relative to networks with 100 nodes and are highlighted in red. Growing networks are measured once every 100 nodes are added. Simulated networks range from 100 up to 1500 nodes. Mean values are averaged for all nodes in a graph and across 100 independent realizations.

Figure 3 reports on the mean closeness centrality and distance entropy of growing BA network models. Network growth follows a preferential attachment process where one node and *m* links are added at every time step [1].

Since it is already known that the average network distance *l* grows as logN/loglogN in growing BA networks with *N* nodes, then it is expected for closeness centrality to decrease with the growing number of nodes. When the number *m* of links added at each time step is smaller than 8, a monotonic decrease in distance entropy is registered during network growth. Instead, when m=8, smaller BA networks display a peak of distance entropy for intermediate sizes (N≈600). Entropy *h* decreases at later steps when more nodes and links are added. We link this decrease in distance entropy with the emergence of hubs due to preferential attachment in larger BA networks. Hub nodes tend to have distance 1 from a significant fraction of nodes in the network, thus considerably lowering the average distance entropy.

## 4. Cartography Based on Distance Entropy and Closeness Centrality

In the previous sections, we focused on characterising the mean distance entropy of networks by considering different models. We now focus on the structural patterns of individual nodes that emerge by considering closeness centrality and distance entropy together in one given network. We consider distance entropy as an estimator of the variation of distances of a given node from all its neighbours, thus providing additional information compared to considering closeness centrality only (which reports only on the mean distance of a node from the other nodes).

We combine information from both the closeness and distance entropy of a node by introducing the concept of *distance entropy cartography*. We draw inspiration from the concept of cartography introduced by Guimerá and Amaral for characterising the role played by individual nodes in community structure [29], a concept later generalised to the participation of nodes on multiplex structures [30]. Network cartographies are useful for visualising the map of topological patterns that nodes have in a given network structure.

**Figure 4:**
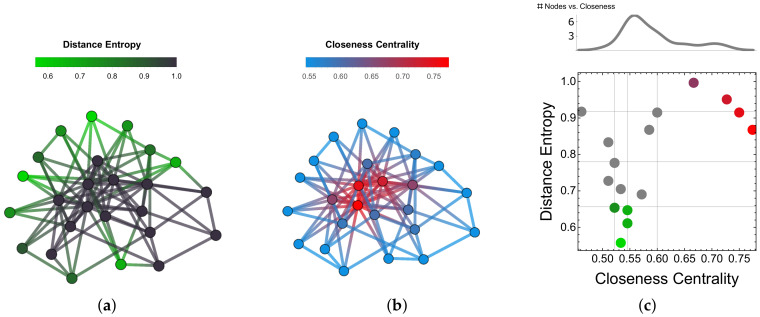
Distance entropy provides different centrality information on nodes, compared to closeness centrality. Here we consider a BA network with N=25 nodes and m=4. (**a**) Nodes with low distance entropy are highlighted in green. (**b**) Nodes with high closeness are highlighted in red. (**c**) Cartography representing the distance entropy and closeness centrality of individual nodes in the network. Gray lines indicate quartiles. Nodes with the lowest (highest) distance entropy (closeness) are highlighted in green (red). The two sets of nodes do not overlap. Not considering distance entropy would lead to a closeness distribution reported in the top subpanel, where many nodes would end up displaying similar closeness centrality despite their different connectivity patterns, here highlighted by their distance entropy.

In Figure 4 we show an example of distance entropy cartography for a toy model (BA network with m=4 and N=25 nodes). Sub-panel (a) highlights nodes with the lowest distance entropy (i.e., a more homogeneous distribution of network distances) while (b) highlights nodes with the highest closeness centrality (i.e., at shortest average distance in the network). Note that nodes with higher closeness centrality also tend to have higher distance entropy, as they are closer to each other but further apart from peripheral nodes. Consequently, the set of nodes with high closeness does not overlap with the set of nodes with low distance entropy—the two metrics provide complementary information. We define a cartography by a 2D space where each node has its distance entropy and its closeness as coordinates (sub-panel (c)). Representing nodes in this 2D space is informative; in fact, most of the nodes in the network have similar closeness centrality around $c_{i}=0.55$ (see also sub-panel (c), top plot) but display evidently different values of distance entropy, ranging from h=0.53 up to h=0.84. Hence, considering distance entropy can help to reduce the degeneracy observed when considering closeness only: nodes having similar closeness centrality are found—by means of the distance entropy—to be differently connected to the rest of the network.

## 5. Applying Distance Entropy Cartography to Multiplex Lexical Networks

We apply the cartographic analysis previously introduced to multiplex lexical networks [6,31], successfully applied for modelling trends of progressive language impairments such as aphasia [19], but also patterns of language development such as modelling and predicting strategies of word learning in toddlers [18]. When considering multiplex lexical networks and learning, we wonder if distance entropy can provide any improvement for detecting word learning strategies in toddlers.

Here, we use the same empirical networks used in [18] (i.e., multi-layer edge-coloured networks where nodes represent words). There are no explicit inter-layer links, and layers represent semantic relationships (e.g., “dog” and “cat” share the feature of being an animal) and phonological similarities (e.g., “bad” and “bed” differ by one phoneme only) among words. For ranking words in the order they are learned by most English toddlers between 18 and 30 months of age, we use longitudinal data from the CHILDES dataset accessed through TalkBank [32]. The longitudinal data allows the reconstruction of the fraction of children producing a certain word in a given month (i.e., production probability). Within each month, words are ranked in descending order of production probability. This ranking represents a proxy for the normative learning of most toddlers [18,33,34].

Recently, different network approaches have been successfully used for predicting the acquisition of words based on their network features (e.g., word degree, closeness centrality, network gaps) [18,33,35,36]. Here, we rank words according to the introduced cartography and then compare against the ranking of the estimated age of acquisition, in which words acquired earlier (e.g., “mommy”) are ranked higher than words learned later (e.g., “picture”). The extent to which an artificial ranking $r_{a}$ predicts the words learned according to the normative learning ranking $r_{l}$ is measured through the word gain:

(5) $$g\left(r_{a},t\right)=\frac{O\left(r_{a},r_{l},t\right)-R\left(r_{l},t\right)}{t},$$

representing at position *t* the fraction of words predicted as *correctly learned* in $r_{l}$ by the network ranking $r_{a}$ ($O(r_{a},r_{l},t)$), with respect to random guessing ($R(r_{l},t)$). A word gain of 20% when t=200 words have been learned means that $r_{a}$ predicts as correctly learned 40 words more than random ranking.

**Figure 5:**
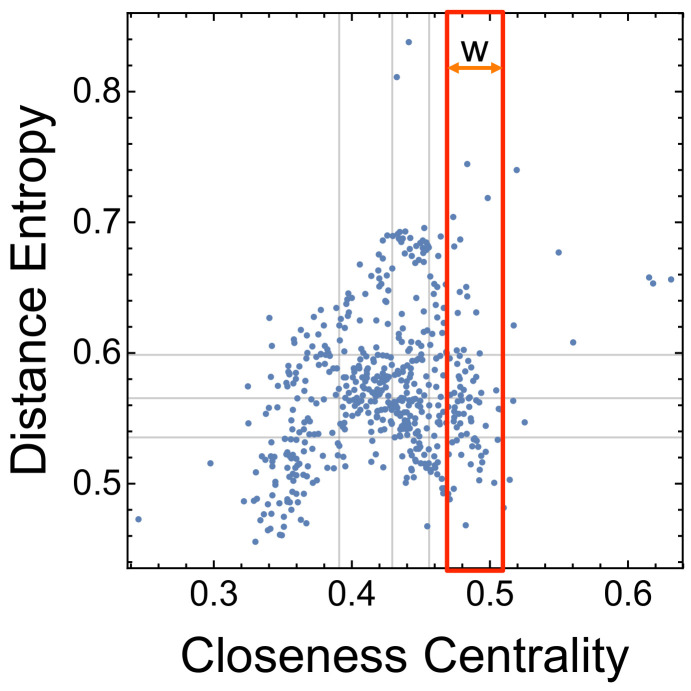
Distance entropy cartography for the N=529 words in the multiplex lexical network of young toddlers. Within a window of width *w*, nodes with similar closeness centrality can have quite different distance entropies.

Multiplex closeness centrality provides a word gain higher than other measures (e.g., betweenness, degree, and local clustering coefficient) on both single and multiplex network topologies [18]. Hence, here we focus on the ranking $r_{clo}$ of descending closeness centrality and use it as a reference level to test whether enriching it with information from distance entropy can achieve higher word gains. Distance entropy is computed on the multiplex shortest paths, namely the shortest paths where links from different layers can be combined together [3]. The resulting cartography is shown in Figure 5.

The distance entropy cartography indicates that many nodes with similar closeness centrality differ highly in their distance entropy. We quantify this notion of closeness similarity by considering nodes having closeness around $c^{*}$ within an interval $[c^{*}-w,c^{*}+w[$. Here *w* represents an interval width, a tolerance parameter determining which nodes have closeness similar to $c^{*}$ up to a value *w*. If w=0, then our definition of similarity would reduce to identifying ties (i.e., considering as similar nodes having the same value of closeness $c^{*}$). Provided its interpretation in terms of similarity, we consider values w≪1.

We use the cartography and closeness similarity for building artificial rankings of words based on both closeness centrality and distance entropy. Starting from the maximum value of closeness $c_{max}$ in the network, we build bins $b_{i}=[c_{max}-\left(i+1\right)\cdot w,c_{max}-i\cdot w[$. A ranking $r_{e}\left(w\right)$ is produced by ranking all nodes in bin $b_{i}$ in increasing order of distance entropy. Within each bin $b_{i}$ we rank nodes from lower to higher distance entropy because the lower $h_{j}$, the more a node *j* is connected to all other nodes in the network. Hence, nodes with lower distance entropy are expected to be more central in the network. Note that distance entropy provides different information compared to other multiplex centrality measures such as multidegree [3] or PageRank versatility [37], since the induced node rankings overlap with distance entropy only for 30% (Kendall Tau τ=0.30%) and 21% (Kendall Tau τ=0.21) respectively.

Note also that when *i* increases the average closeness of the considered nodes decreases, so $r_{e}\left(w\right)$ is a rank in which: (i) highest closeness nodes are on average ranked higher; (ii) nodes with closeness similar up to a tolerance *w* are ranked according to their distance entropy. Hence, in $r_{e}\left(w\right)$, words in the left-lower part of the entropic cartography are ranked higher (i.e., words with high closeness and low distance entropy).

This ranking is a function of the window *w*: when w=0, then distance entropy has no effect and $r_{e}\left(0\right)$ is equivalent to ranking nodes in descending order of closeness ($r_{clo}$); when w=1, then all the nodes are ranked according to their distance entropy and closeness plays no role in affecting the ordering. We investigate the influence of *w* in providing ranks mixing distance entropy and closeness centrality for improving prediction performances (i.e., increasing the average word gain). We focus on the early stages of cognitive development between months 20 and 23, which are called the Early Learning Stage in which multiplex closeness centrality provided the highest word gains.

We measure increases or decreases in prediction power of which words are learned early by toddlers by considering a relative word gain improvement:

(6) $$\Delta g\left(r_{e}\left(w\right),t\right)=\frac{g\left(r_{e}\left(w\right),t\right)-g\left(r_{clo},t\right)}{g(r_{clo},t)}.$$

A relative word gain improvement $\Delta g(r_{e}\left(w\right),150)=0.1$ means that when 150 have been learned, the ranking considering together closeness and distance entropy predicts as correctly learned 10% more words than considering closeness only. Provided that improvements depend on the value of *w*, a scan of different values is essential. For each value of *w*, we compare the observed improvement against a distribution of random improvements obtained by fixing the same *w* and the same bins but ranking words at random rather than according to their distance entropy. These randomised ranks represent our null models and allow one to quantify the statistical significance of word gain improvements observed when using the cartography.

Table 1 reports the word gain improvements averaged between months 20 and 23 (the Early Learning Stage) for different values of *w*. When 0<w≤0.05, positive improvements in word gain are registered, while for w>0.05 only negative improvements are retrieved. The registered positive improvements are statistically significant at a 0.01 significance level when 0.015≤w≤0.025, indicating the presence of a window where learning high closeness words with low distance entropy leads to marked improvements in predicting which words are learned by toddlers. In such cases, the average word gain achieved with the entropic cartography is +13.3%.

How do the results of the distance entropy cartography compare against other centrality measures? In order to test the importance of distance entropy, we binned nodes in terms of decreasing closeness centrality but ordered them in decreasing order of multidegree centrality within bins of the same width 0.015≤w≤0.025 from the distance entropy cartography. Using degree rather than distance entropy remarkably worsened prediction performances, as it produced on average only negative improvements of word gain (≈−15%).

These results indicate that considering distance entropy on top of the multiplex closeness centrality is beneficial for achieving better predictions of the way most English toddlers learn words. All in all, this application to real-world networks indicates that the topological information encapsulated in the distance entropy can provide additional insights in discovering and interpreting patterns of real-world complex systems.

## 6. Discussion

In this paper, we introduce a new type of distance entropy, characterising the distribution of path lengths from a given node in a network. Compared to previous important results in the field [20,21,22,23], our main novelty is in considering the entropy of paths of all lengths at local and global levels. We provide analytical results for the distance entropy of individual nodes in regular graphs and show that it is minimum when a node is adjacent to all other nodes in the network. From the analysis of the mean distance entropy in well-known network models such as ER random graphs, BA scale-free networks, and small-world networks, we observe that the creation of links either uniformly at random or through preferential attachment generally decreases the heterogeneity of path lengths, decreasing distance entropy. There is a noticeable exception in ER random graphs, where we observe a tipping point for distance entropy in increasingly denser ER random graphs. We attribute this change to the sudden emergence of short-cuts in the system. No tipping points have been detected in small-world and BA scale-free networks.

Note that our definition of distance entropy considers only shortest paths, in contrast to other information-theoretic network measures based on network distance such as the centrality information introduced in [38] or the resistance centrality from [39]. Both these quantities strongly correlate with closeness centrality, since they are mainly variations of this measure, at variance with the distance entropy measure proposed in this work.

In fact, we provide evidence that distance entropy carries different topological information compared to other measures based on distance, such as closeness centrality. Note that closeness is combined with distance entropy because they are both relative to shortest path lengths: closeness is the mean of the distribution of path lengths, while distance entropy is related to its variance (i.e., maximum entropy indicates paths of all possible path lengths, minimum entropy indicates null variance). Consequently, because of this strong tie, we focused mainly on closeness and distance entropy for defining the distance entropy cartography to better characterise nodes’ centrality in complex networks. The additional information carried by distance entropy allows one to further distinguish nodes with equal (or very similar) closeness centralities, thanks to the fact that such nodes can be more or less heterogeneously distant from the rest of the system.

In the current study, the concept of closeness similarity has been parametrised by means of a parameter *w* representing the tolerance up to which two nodes are considered having similar closeness centralities. We did not fix *w* in the current analysis in order to prevent overfitting, however its interpretation as a tolerance indicates that w≪1. Additional criteria from statistics such as using percentiles or data clustering techniques could be pursued in future work.

We use the information combined by the cartography to rank words in multiplex lexical networks [6,18,19] and to predict the order with which toddlers learn words during cognitive development. We show that resolving the degeneracy of nodes with similar closeness centrality but (very) different distance entropy provides consistently positive improvements in predicting word learning strategies by at least 13%. Although these improvements might seem small, two additional elements must be considered. Firstly, multiplex closeness centrality is already an optimal measure of word prediction, in the sense that it vastly outperformed single- and multi-layer versions of degree, betweenness, local clustering, PageRank, and eigenvector centralities in early word prediction, so that improvements to its prediction performances are remarkable. Secondly, distance entropy provides positive improvements that are not captured by other network statistics such as degree, which conversely provides negative word gain (≈−15%) for the same values of *w*. This result underlines the importance of considering distance entropy.

Our results provide evidence that English-speaking toddlers mainly tend to acquire words with high closeness centrality and low distance entropy early on during language acquisition. These words display less heterogeneous distributions of path lengths, with smaller central moments, on the whole multiplex lexical structure, and are thus easier to reach from other words in the mental lexicon. From the perspective of the cognitive sciences, the improvement in the prediction of word learning indicates a cognitive advantage in learning words more central for the spread of information within the mental lexicon of word-similarities. This finding is in agreement with other independent studies indicating that closer words in linguistic networks are more easily identified in healthy subjects [17] and produced in subjects with aphasia [19]. Note that a drawback of our modelling approach is that it does not account for individual differences in word learning, but rather refers to the average trend at the population level of healthy English-speaking toddlers. Quantifying the powerfulness of distance entropy cartography for predicting the word learning of individuals requires additional data, and it poses an interesting direction for future research.

From a network perspective, distance entropy provides different topological information compared to closeness centrality, but the two measures share the same computational cost. Hence, the proposed cartography can also be used for investigating large-scale networks, providing an important tool for the investigation of structural patterns in real-world networks where the distances among nodes matter.

## Figures and Tables

**Table 1:** Improvements in word gains (relative to the reference closeness case) for different values of binning width *w*. *p*-values are relative to the observed improvement relative to a reference distribution. Reference distributions are obtained by ranking nodes at random (rather than through distance entropy). When w>0.05, no improvements are obtained.

Width *w*	Improvement (%)	*p*-Value
0	0	1
0.005	+3.9%	0.3
0.010	+7.9%	0.05
0.015	+13.1%	0.001
0.020	+13.3%	0.001
0.025	+13.6%	0.001
0.030	+7.9%	0.01
0.035	+8.0%	0.01
0.040	+4.0%	0.03
0.045	+5.1%	0.01
0.050	+0.1%	0.01
